# Transforming nursing work environments: the impact of organizational culture on work-related stress among nurses: a systematic review

**DOI:** 10.1186/s12913-024-12003-x

**Published:** 2024-12-02

**Authors:** Evans Kasmai Kiptulon, Mohammed Elmadani, Godfrey Mbaabu Limungi, Klara Simon, Lívia Tóth, Eva Horvath, Anna Szőllősi, Dahabo Adi Galgalo, Orsolya Maté, Adrienn Ujváriné Siket

**Affiliations:** 1 Doctoral School of Health Sciences, Faculty of Health Sciences, University of Pécs, Pécs, Hungary; 2 Director of Foreign Affairs, PTE Faculty of Health Sciences, Doctoral School of Health Sciences, University of Pécs, Pécs, Hungary; 3 Department of Nursing and Midwifery, Head of Masterster’s Programmeogramme, Doctoral School of Health Sciences, President College of Nursing, University of Debrecen, Debrecen, Hungary

**Keywords:** Nursing, Organizational culture, Stress mitigation, Systematic literature review, Work-related stress, Work environment

## Abstract

**Background:**

Creating a healthy and conducive nursing work environment is a universal global nursing concern. Work-Related Stress, global nursing and a public health problem that has continued to bedevil the world healthcare systems is of a particular interest. It has not only compromised the quality of patient care but also negatively impacted nurses’ quality of work life and adversely affected global healthcare management. Organizational culture is an important determinant of nurses’ work-related stress, yet it remains systematically under-researched. Despite a plethora of research on work-related stress in nursing environments, there are few dedicated systematic literature reviews, and this study aimed to fill this gap.

**Objective:**

To determine the scientific evidence in the literature, on the impact of organizational culture on work-related stress among nurses and provide valuable insights to mitigation of work-related stress among nurses.

**Design:**

A Systematic literature review.

**Methods:**

The review followed the Preferred Reporting Items for Systematic Reviews and Meta-Analysis guidelines. A comprehensive literature search was conducted in major electronic databases, including PubMed, Scopus, Web of Science, Ovid Medline, Embase and CINAHL in July 2023. Studies that met the inclusion criteria set were screened using Rayyan and Covidence. The Mixed Methods Appraisal Tool (MMAT) was used to assess the quality and risk of bias.

**Results:**

The search generated a total of 2,113 records, and 13 were included. Thematic analysis generated three main themes: types of organizational culture, organizational climate, and organizational politics, each with distinct effects on nurses’ work-related stress. We found overall that, positive organizational culture, positive organizational climate and positive organizational politics were consistently associated with nurse’s happiness and joy at the workplace and lower levels of work-related stress while negative organizational culture, climate and politics were strongly associated with nurses’ work-related stress.

**Conclusions:**

This review underscores the crucial role of organizational culture in nursing work environments and its impact on nurses’ stress levels, offering valuable insights for the mitigation of work-related stress and the transformation of the nursing profession.

**Supplementary Information:**

The online version contains supplementary material available at 10.1186/s12913-024-12003-x.

## What is already known

- Work-Related Stress is a major nursing and public health problem globally.
- Organizational culture plays an intricate, causative, and preventive role in shaping and intensifying work-related stress.
- Organizational Culture is a complex terminology that has long been in use in business and military studies, however little attention has been put in nursing and health.

## What this paper adds

- This systematic review examined organizational culture in nursing and found that, positive organizational culture, organizational climate and organizational politics were consistently associated with reducing nurse’s work-related stress while negative increase work-related stress.
- The review underscores the power of organizational culture in transforming the nursing work-environment and mitigating work-related stress.

## Background

Nurses serve as the cornerstone and fundamental human resource within healthcare systems, playing a critical and integral role in shaping the health landscape of the global society. Therefore, paying attention to, and prioritizing their health and well-being will guarantee the health of the entire world [1]. In the race to safeguard nurses’ health and enhance quality nursing care for the world’s populace, Work Related Stress (WRS) has long been identified as one of the most significant risk factors in the workplace, adversely affecting the health and well-being of nurses [2].

In the contemporary global healthcare systems, one of the major nursing and public health concerns for nurses revolves around WRS, which emanates from the prevailing organizational culture within their work environments [3–5]. WRS, also known as job stress or occupational stress, is defined by The World Health Organization (WHO) as “A response people may have when presented with work demands and pressures that are not matched to their knowledge and abilities and which challenge their ability to cope” [6]. NIOSH (National Institute for Occupational Safety and Health) characterizes this definition further by defining it as “the harmful negative physical and emotional reactions that occur when the requirements of the job do not match the worker’s talents, capabilities, resources, or needs” [7, 8]. Other major international organizations that have recognized WRS as a critical public health and nursing problem include; the International Labour Organization (ILO) [9], International Council of Nurses (ICN) [10], the European Agency for Safety and Health at Work (EU-OSHA) [11], the International Commission on Occupational Health(ICOH) [12], Scientific Committee of Occupational Health Nursing [13], the American Nurses Association (ANA) [14] and the Federation of Occupational Health Nurses within the European Union (FOHNEU) [15]. Collectively, these organizations have adopted unequivocal stance: that WRS constitute a significant workplace hazard with far-reaching implications for the health, safety and well-being of the global nursing and other healthcare workforce, necessitating immediate and decisive preventive actions.

Numerous factors have been documented as contributing to the WRS among nurses. One of these major causes is Organizational Culture (OC) [5, 16, 17]. Other causes that also mirror around and are largely part of OC, include: understaffing, lack of resources to work, lack of job control, failure of organizations to involve nurses in decision making, inadequate recognition and compensation, inadequate training and lack of continuing education and training opportunities to keep nurses abreast with new technologies, treatment uncertainty, burnout, poor collaboration at the workplace, job ambiguity and lack of role clarity, lack of professional autonomy, role conflict, and a lack of support from supervisors and coworkers [18–21]. Moreover, workplace bullying, violence and incivility from patients, patient relatives, co-workers, doctors, administration, alongside racism, particularly targeting internationally educated and migrant nurses, worsen nurses’ work-related stress [22–24]. In addition, job insecurity, job instability, and layoffs especially when countries face economic crises put overwhelming stress on nurses [1]. Furthermore, nurses’ work involves long working hours with irregular shifts, mandatory overtime and night shifts which disrupt nurses’ sleep patterns and personal lives [25, 26]. Nursing is emotionally and psychologically demanding [27]. In various workplace settings, nurses encounter a diverse range of patients, from those who are happy to those who are suffering, experiencing emotional distress, or facing life-threatening situations. This can be emotionally taxing especially when nurses are exposed to traumatic and life-threatening events. In addition to this, nurses are overworked and play other non-nursing roles coupled with heavy paperwork documentation and demands to comply with filling patient Kardex’s and nursing care plans [28]. Non-nursing chores and difficult work situations for example manual filing and documentation exacerbate work-related stress. Occasionally, nursing care has been compared to the manual construction industry [26], due to heavy lifting and turning of patients and long-standing hours that drain nurses and cause musculoskeletal problems [29].

WRS has detrimental effects on nurses’ health and organizations’ abilities to provide quality nursing care. Mentally, it can lead to mild effects like insomnia, anxiety, and emotional exhaustion, to major effects like panic attacks, depression, burnout, and mental disorders [3, 30]. WRS weakens nurses’ immunity predisposing them to gastrointestinal diseases (stomach ulcers, heartburns, and indigestion) [1], several cardiovascular diseases, musculoskeletal disorders, back pain, headache, obesity, and other chronic diseases including hypertension [18]. Previous researchers have also alluded that work-related stress increase nurse turnover, job dissatisfaction, work disengagement, medical errors, nurse absenteeism, organizational cynicism, alcohol, and drug abuse and reduces job performance [31–34]. It can be concluded that, a stressed nurse is a dangerous nurse, especially, when stress causes nurses’ cognitive impairment, hence leading them to make grave medical errors. WRS can also spill into nurses’ personal lives causing poor social life outside the workplace including strained relationships, social isolation, suicidal thoughts and even death [8].

WRS is not only costly to the health of nurses or the organization’s ability to provide quality care but also to the global economy. A systematic review of economic costs of work-related stress from 15 studies; Australia, Canada, Denmark, Sweden, United Kingdom, and other countries of the European Union estimates that WRS economic losses range from US$221.13 million to $187 billion annually [35]. American Institute of Stress, statistics 2023, claims that about 1 million Americans miss work every day due to stress and this situation is never any better in any continent of the world. Stress, anxiety, and depression cost the global economy over US$ 1 trillion annually in lost productivity [36]. Stress is a primary cause of 80% of all occupational injuries and 40% of the financial burden in the workplace [37].

### Organizational culture and work-related stress in nursing and healthcare

“Organizational Culture” (OC) is a complex terminology that has been the subject of many definitions by researchers and scholars. Rob Goffee and Gareth Jones in their book “*The Character of a Corporation: How your company’s culture can make or break your business”* alludes that OC is “simply how we do things around here” [38]. It is the personality of an organization and at its best, it can make people feel good about their working environment and at its worst, it can make people feel fearful, frustrated and broken down.

OC is a management concept that places leadership at its core [39, 40], and it’s said that leadership and management are the two sides of the same coin [41].

For this review, we considered the definitions of OC as “the way things are done within hospital organizations”. It includes rules, policies, mission, and strategy, assumptions, values, workplace morals, beliefs and shared expectations, organizational leadership and management, organizational structure among other aspects of workplace culture.

Culture acts as a fabric and glue that binds workers together [42]. It dictates how employees view and perceive their organization, leadership, and workplace. Culture is shared and learned over time [39]. It also keeps changing and not constant. Hospitals, big or small have their own OC that influences nurses’ happiness or work-related stress [2]. OC determines how an organization functions and how its members interpret events both within and outside of the hospital [39].

OC has been identified as one of the main sources of stress among nurses [2, 43]. Majority of work-related stressors: Understaffing, lack of autonomy, inadequacy of resources, lack of leadership support, lack of opportunities for studies, lack of nurses recognition, punitive rules, toxic work environment characterized by condonement and tolerance of bullying, incivility and lack of respect to nurses are problems relating to OC [17, 22, 44]. A culture that doesn’t encourage open communication, mentoring, or peer support can lead to feelings of isolation and anxiety [45]. Rules, policies, standards procedures, and regulations form part of OC [46]. Frequent changes in policies and procedures or a lack of clear, standardized guidelines can lead to confusion and stress among nurses [47]. They may constantly worry about making errors or facing disciplinary action due to inconsistent expectations [48, 49].

Researchers have also elucidated those organizational cultures with unrealistic expectations, such as demanding high productivity and flawless performance without providing the necessary resources and support, can lead to chronic stress and a sense of inadequacy. Favouritism, preferential treatment, unfair treatment, biased reward systems on promotion, employment, job confirmation or any other discrimination based on ethnicity, tribalism, race, colour, religion, sexual orientation, cadre, job group etc., impose stress on employees [24].

Despite this critical role that OC plays in nurses’ WRS, there is a paucity of dedicated comprehensive systematic literature reviews. Nursing OC data in the literature is fragmented, and most studies on this topic are limited to specific health contexts not work-related stress, and sectors other than nursing. Therefore, this rigorous systematic review is needed to fill this gap and update the evidence.

### Aim of this systematic review

This systematic literature review aimed to evaluate the scientific evidence available in the literature, on the impact of OC on WRS among nurses and provide valuable insights to mitigation of WRS in nursing.

## Methods

### Review question

- What is the impact of organizational culture on WRS among nurses?
- Based on the existing evidence, what recommendations focusing on organizational culture can be derived to transform nursing work environments and mitigate work-related stress?

### Study design

This study was a systematic review. Before it was conducted, a priori protocol for the research was registered in PROSPERO with registration identification number CRD42023440170. The methodology used in this review was based on the Cochrane Handbook for Systematic Reviews of Interventions [50] and The Preferred Reporting Items for Systematic Reviews and Meta-Analysis (PRISMA) flow chart [51] was used to present the search strategy.

### Search strategy and selection criteria

**Fig. 1:**
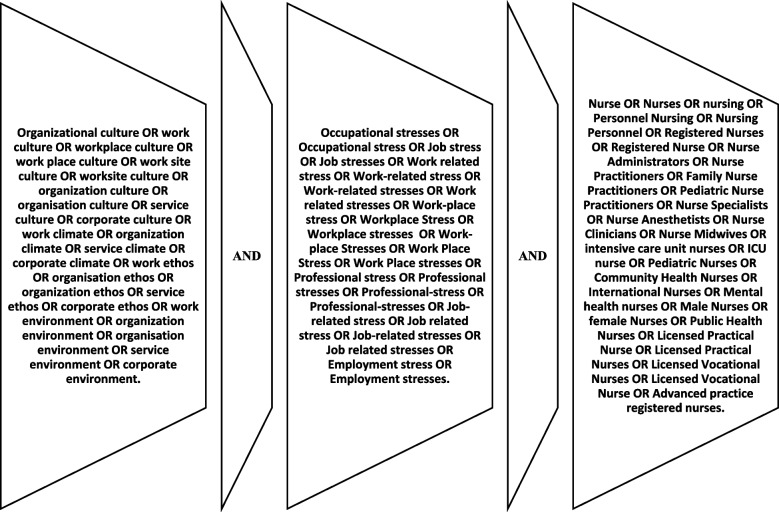
Search strategy

To find published articles included in this study, a comprehensive literature search was conducted in July 2023 in the following databases: Ovid Medline, PubMed, Embase, Scopus, CINAHL and Web of Science. The research question followed (PEO) framework [52] which stands for (P)-population(nurses), (E)-exposure (organizational culture), and(O)-outcome (work-related stress). The search method employed the Boolean operator “OR” between key synonym words, which were then linked with “AND” to complete the search as shown in Fig. 1 below.

### Inclusion and exclusion criteria

All studies had to meet the following criteria to be included: be from any country and conducted on nursing personnel of any cadre as the study population, be original articles in English, used any study design, no limitation to the year of publication, WRS was the outcome of the study, and the stress was related to OC or OC variables. We excluded conference papers, other systematic literature reviews, meta-analyses, letters of opinion, editorials, essays, case studies, and literary works. Additionally, we did not consider comments or narrative articles, as well as full-text articles written in languages other than English and paid studies. Furthermore, studies without nurse participants or those involving a mixed group of nurses and other professionals were also excluded.

### Screening of the articles

The database search results were entered into EndNote to collect all together and subsequently exported into the Cochrane software for deduplication and screening. Blinding was done, and working independently authors EKK, ME, GML, KS, LT, EH, AS and DAG conducted tittle and abstract screening then full text screening to evaluate each study for eligibility inclusion. Disagreements on the articles were resolved through discussion and consensus during screening. In situations where there was no agreement reached, a third screener was involved.

### Data extraction and data synthesis

The following data from the included studies was extracted into a standardized Excel sheet for analysis: the primary author, year of publication, the country in which the research was conducted, the main study objective/aim, study design, sample size, study population, study setting, response rate, exposure details (organizational culture type or variable), the measurement instruments employed, outcome details (work-related stress), the measurement instrument measure, and the significant findings. Tables were created to organize the information. The data synthesize was thematical, qualitative and narrative.

### Quality assessment of included studies

The included studies were assessed by EKK and ME for quality using The Mixed Methods Appraisal Tool (MMAT) [53]. This is a critical appraisal tool that is used to appraise studies of various methodologies together. It has five major sections numbered (1) qualitative research, (2) randomized controlled trials, (3) nonrandomized studies, (4) quantitative descriptive studies, and (5) mixed methods studies, with each section having five questions to appraise the methodological quality of studies. Responses to appraising questions are given in the form of “Yes’ ‘No’ or ‘Can’t tell’. “Yes” is scored 1 or one star (*), which represents twenty per cent quality, and ‘No’ or ‘Can’t tell’ both are scored 0, which is (0%) quality. The scores are averaged to find the overall quality of the paper. Articles that averagely scored above 60% were included and reported in this review.

## Results

### search outcomes

**Fig. 2:**
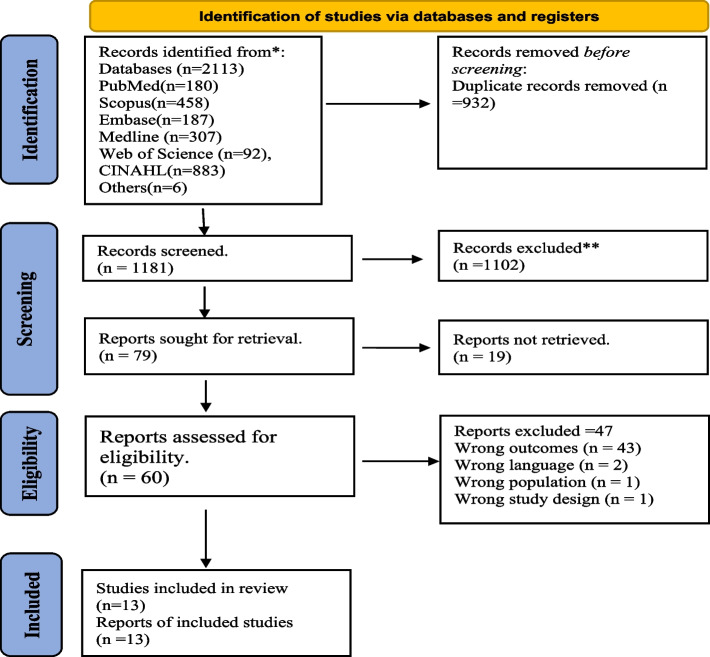
PRISMA flow diagram illustrating search results for the systematic review

Our search from all the databases yielded a total of 2,113 articles. Nine hundred and thirty-two duplicates were removed, and 1,181 studies were screened for title and abstract out of which 1102 were further excluded and 79 sought for retrieval. Sixty studies went through a rigorous full-text screening screened for and we finally included 13 studies for this review (see Fig. 2*. PRISMA flow diagram*). These studies were appraised for methodological quality using the MMAT tool and they all met the average 60% quality inclusion criteria set. Two studies scored 60%, while two others scored (80%). The rest of the articles scored 100% indicating that their methodological quality was high (*See supplementary table S1).*

### Characteristics of the included studies

#### Language, location, design and setting

All the 13 included studies were written in English language and were all conducted in hospital settings. Almost half of the studies were done in European countries Belgium (1), Greece (1), Hungary (1), Finland (2) and Turkey (1). The rest of the studies were done in Iran (3), Egypt (1), USA (1), South Korea (1) and Australia (1) (*see *Table 1*. Characteristics of the included studies).* The majority of the studies (12) employed quantitative and cross-sectional study designs while only one [40] used qualitative design. The studies had a combined study population of 3,813 nurses. The study with the least response rate was 65% while highest was 100%.

**Table 1:** Characteristics of the included studies

NO	**Author, year & country**	**Design**	**Population(N) &** **response rate**	**Setting**	**Work-related stress reported**
1	Papadionysiou E, et al., 2022. Greece [54]	Cross sectional	269 general Nurses,81.5%	Hospital	Structural Equation Modelling. Stress levels not reported
2	Moustafa Manal Saleh et al., 2015. Egypt [55]	Descriptive correlation	315 general nurses, NR	University Hospitals	88.3% of the nurses had high-stress levels
3	Atabay et al., 2015. Turkey [56]	Cross sectional	201Registered Nurses,72%	Hospital	On the Likert scale, 6 indicating high distress, organizational constraints were (M = 5.03, SD = 0.68), lack of sufficient time and resources (M = 5.22, SD = 0.74) & misinformed and over-treated patients (M = 4.59, SD = 0.98)
4	Van et al., 2014. Belgium [57]	cross-sectional survey	365 Nursing unit managers,68%	Hospital	One out of six nursing unit managers had high to very high feelings of emotional exhaustion
5	Siket et al., 2020.Hungary [58]	cross-sectional Study	367 General nurses,73.4%	Hospitals	On a Likert scale, 4 indicating the highest stress. Mean stress was 16.71 out of a maximum 40 indicating low stress
6	Shirey et al., 2009. USA [40]	Qualitative descriptive	21 Nurse managers, 100%	Acute care hospitals	Of the 21 nurse managers,43% (9) worked in stressful and unhealthy work environments
7	Movahedi et al., 2020.Iran [59]	Descriptive, analytical	280 Licensed registered nurses,93.3%	University Hospitals	The Nursing Stress Scale was used to measure WRS. However, the levels were not clearly reported
8	Lee et al., 2020. South Korea [5]	Cross-sectional	252 Registered nurses,98%	Hospitals	The average stress mean out of 100 was 47.48, with SD 9.89 indicating moderate stress
9	Hayes et al., 2015.Australia [60]	Cross-sectional online survey	417 Haemodialysis nurses,78%	Haemodialysis units, Hospital	Nursing Stress Scale, measured in Likert with 4 indicating highest stress, the overall mean of NSS was 2.08 indicating somewhat low stress
10	Hahtela et al., 2015. Finland [61]	Cross-sectional descriptive	220 General nurses, 65%	Primary healthcare organizations	On a Likert scale, 7 indicating the highest stress level, the overall mean score for stress was low [mean = 2.75; standard deviation (SD) 0.66] indicating low-stress levels
11	Habibzadeh et l., 2020.Iran [62]	Survey	298 General nurses,90.3%	Teaching hospitals	Structural Equation Modelling. Stress levels were not reported but burnout in the study was reported to be high. psychological distress had the highest mean score
12	Eskola et al., 2016, Finland [63]	Quantitative cross-sectional	96 Operation room nurses, NR	Hospital	On Likert scale,7 indicating the highest stress, the overall stress was low (M = 2.3)
13	Aghaei et al., 2020. Iran [1]	Descriptive cross-sectional	712 General nurses,89%	Hospital	Structural Equation Modelling. Stress levels not reported

#### Work-related stress results

Two studies [55, 56] reported very high levels of WRS while four studies [57, 58, 61, 63] reported low-stress levels. Studies that reported moderately high levels of WRS were [5, 40, 60]**.** Three studies in data analysis used structural equation modelling [1, 54, 62] and did not clearly report the levels of work-related stress as well as [59] (see Table 2*. summary of the findings*)*.*

**Table 2:** Summary of the findings(*n* = 13)

Author, Year, Country	Objective of the study	Measurement tools used	Key findings
Papadionysiou E, et al., 2022. Greece [54]	To investigate the prevailing organizational culture in Greek hospitals and its connection to employees’ work attitudes, including work engagement, job satisfaction, and job burnout	-The Organizational Culture Assessment Instrument (OCAI) subscales (clan, market, hierarchy, and adhocracy)	The prevailing culture was hierarchy and clan. Organizational culture significantly and positively influenced burnout and had a positive but statistically insignificant impact on work engagement and job satisfaction
Moustafa Manal Saleh et al., 2015. Egypt [55]	To investigate the relationships between organizational culture, occupational stress, locus of control, and personal data among staff nurses	-Organizational Culture Inventory (OCI) sub-scales: constructive, Passive defensive and aggressive defensive cultures. Nursing Stress Scale (NSS)	-Significant majority (88.3%) of nurses experienced high stress level -Effect of organizational culture was not clearly mentioned
Atabay et al., 2015. Turkey [56]	To investigate the relationship between different types of ethical climate and moral distress intensity among nurses in Turkish healthcare settings	-Ethical climate scale-Turkish version -Moral Distress intensity scale with sub-scales	-Stress level was high among the nurses -Negative organizational culture increased nurses’ stress levels
Van et al., 2014. Belgium [57]	Impact of role, job- and organizational characteristics on nurse managers’ work-related stress and well-being	-Leiden Quality of Work Questionnaire for Nurses (LQWQ-N) -Utrecht Work Engagement Scale (UWES) - Maslach Burnout Inventory	-Only 16% of nursing managers had high stress level, while 66.3% reported high to very high work engagement, high Job satisfaction, and low turnover intentions -Positive organizational culture negatively correlated with work related stress
Siket et al., 2020.Hungary [58]	To predict nurses’ intent to stay on the job as a function of organizational culture	-Organizational Climate Scale (OCS) - Rotter Locus of Control Scale (RLC) -Perceived Stress Scale (PSS) -Intention to leave questionnaire	-Stress level was low among nurses - Positive appraisal of organizational climate was negatively correlated with perceived stress, positively with personal self-esteem and doubled the probability of nurses staying on the job
Shirey et al.,2009. USA [40]	To showcase the relationship among authentic leadership, organizational culture, and healthy work environments using a stress and coping lens	Researcher created the tool	43% of nurses worked in stressful and unhealthy negative organizational culture while 57% worked in positive OC -Positive OC had lower stress, high pride & work engagement
Movahedi et al., 2020.Iran [59]	To investigate the relationship between positive perceptions toward Organizational Politics and work-related outcomes in nurses, including stress level	-Perception of Organizational Politics Scale - Nursing Stress Scale (NSS) -Malach-Pines,2005	-Positive organizational politics reduced both stress and burnout
Lee et al., 2020. South Korea [5]	Examine factors affecting clinical nurses’ turnover intention and construct a structural equation model based on the Culture–Work–Health Model	-A Korean version of the Organizational Culture Survey (OCS) - Korean Job Stress scale -Developed questionnaire for Turnover	-Nursing organizational culture had a direct effect on job stress (β = –.70, P = .003) -Organizational culture had indirect effects on turnover intention through job stress and fatigue
Hayes et al., 2015.Australia [60]	To examine the relationships among nurse and work characteristics, job satisfaction, stress, burnout, and the work environment of haemodialysis nurses	-Brisbane Practice Environment Scale (B-PEM; Flint et al. 2010) -Index for Work Satisfaction (IWS; Stamps1997) sub-scales -Nurses Stress Scale -Maslach Burnout Inventory (MBI)	Positive organizational work environment had a positive correlation with job satisfaction and personal accomplishment, while it had negative correlations with job stress, emotional exhaustion, and depersonalization
Hahtela et al.,2015. Finland [61]	To describe nurses’ perceptions of workplace culture, especially regarding stress levels, job satisfaction and the practice environment in primary health care	-The Nursing Context Index (NCI) 78 items Likert scale, with 7 indicating highest positive perception	-The overall mean score for stress was low (Mean = 2.75, SD = 0.66) -Most nurses were unsure if their workplace culture was positive or negative
Habibzadeh et al., 2020.Iran [62]	Examine the mediating effect of the Second Victim Experience (SVE) between safety culture and burnout in Iranian nurses	-Persian version of the hospital survey on patient safety culture (HSOPCS) questionnaire 42 items, Likert scale 5 -Maslach Burnout Inventory (MBI)−22 Items	- Safety culture was negatively related to second victim experience of stress and significantly predicts it (β = − 0.39, p < .01) - Safety culture was significantly related to burnout (β = − 0.24, p < .01)
Eskola et al., 2016, Finland [63]	To investigate the workplace culture in the Operating Room (OR) environment and the factors associated with it (job stress)	Nursing Context Index (NCI) instrument which measures it through 3 key sub-scales: job stress, job satisfaction and practice environment. It’s a 78 Item, 7 Likert scale tool	- low job stress, high job satisfaction and a positive work environment - positive workplace culture slightly and significantly reduced work-related stress
Aghaei et al.,2020. Iran [1]	To investigate the influence of organizational culture on resilience by mediatory effects of occupational stress, job satisfaction, and burnout in nurses using structural equation modelling	-The main factors studied were occupational stress, job satisfaction, burnout, organizational culture, and resilience -Study questionnaire was created from previous validated studies	- Significant positive correlation between organizational culture and job satisfaction (r = 0.29) and resilience (r = 0.21) (P < 0.05). There was a significant negative correlation between organizational culture and occupational stress (r = −0.22), and burnout (r = −0.14) (P < 0.05)

### Thematic Results

**Fig. 3:**
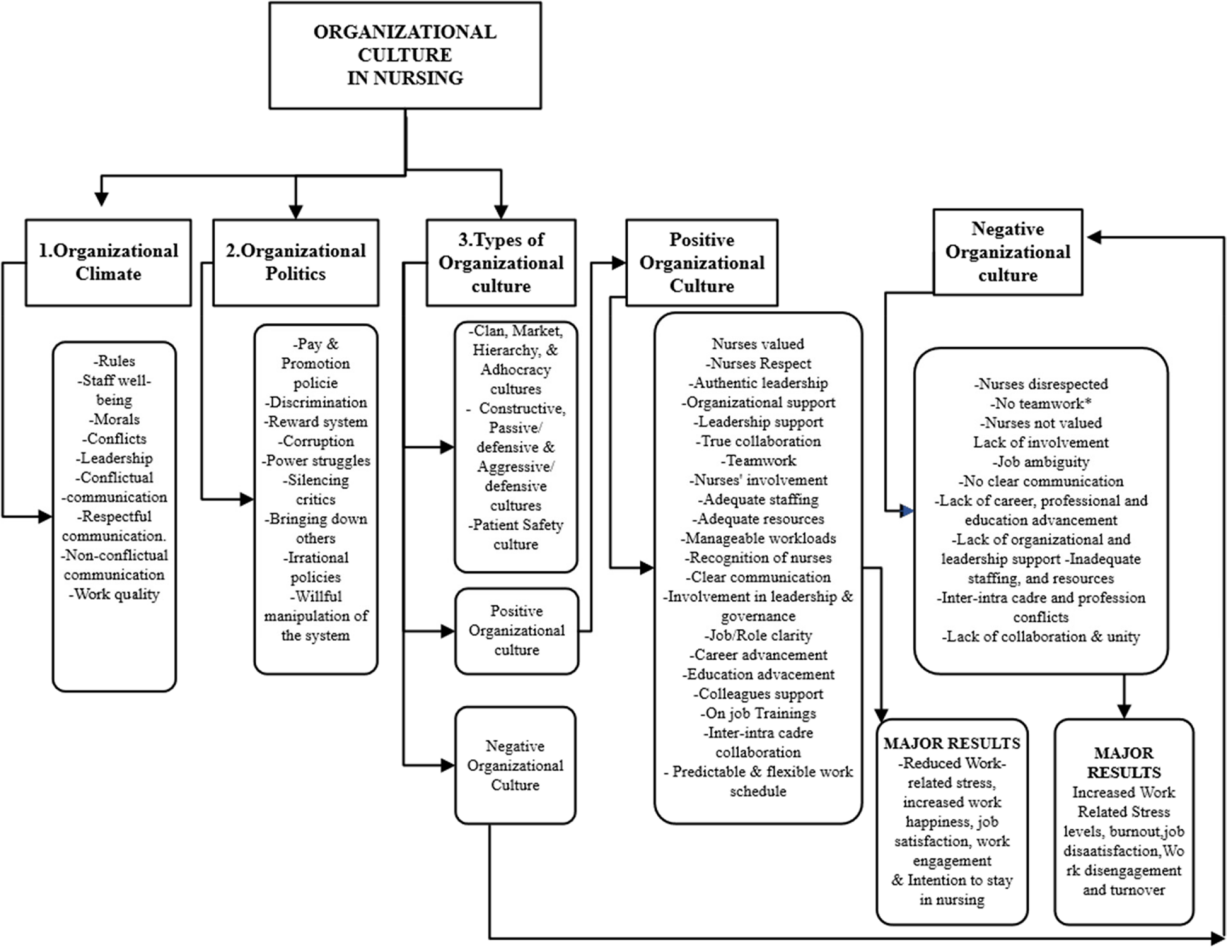
Thematic results for the systematic review

This review investigated OCs that exist in nursing and how they influence nurses’ WRS. The main themes that emerged are (1) Organizational climate as an aspect of organizational culture and its impact on work-related stress, (2) Organizational politics as aspect of OC and impact on WRS and (3) The types of OC in nursing and health care and their impact on WRS among nurses (See Fig. 3 below).

### Types of organizational culture in nursing and healthcare and their impact on WRS

#### Clan, market, hierarchy and adhocracy cultures

Papadionysiou E et al., (2022) classified types of OC into clan, market, hierarchy and adhocracy cultures. *Clan culture* is described as a work culture where nurses are* w*orking together like a big family while supporting one another. This culture values teamwork, trust, respect, and mentoring. Executives mentor, leaders facilitate, and everyone values communication and long-term benefits. *Market culture* is characterized by having leaders and members who are motivated by competition and emphasize productivity and efficiency while *hierarchy culture* prioritizes hierarchy, bureaucracy, efficiency, safe work operations, rule and order while focusing on organizational’s stability. On the other hand, *adhocracy culture* is *d*ynamic and flexible approach to decision-making, encourages innovation, open communication, and decentralized decisions. Leaders are innovators and risk-takers in a creative environment [54].

A study conducted in Greece [54], found that hierarchy culture (59%) and Clan (25%) dominated Greece’s health sector. Work engagement and job satisfaction on a Likert scale 1–5, 5 indicating maximum levels, were high (3.62 and 3.71), but burnout was borderline (2.5). Organizational culture had a positive but statistically insignificant impact on work engagement and job satisfaction, but significantly influenced burnout.

#### Constructive, passive-defensive, and aggressive-defensive

Moustafa Manal Saleh et al., (2014) analysed OC using Organizational Culture Assessment Instrument (OCI®). (OCI®) is an instrument developed by Human Synergistics International and classifies OC into 3 main categories (constructive culture, passive /defensive culture and aggressive defensive culture) each with 4 sub-types drawn in *Synergistics Circumplex. Constructive culture* with sub-cultures (Achievement, self-actualizing, humanistic-encouraging and affiliative) is characterized by behaviours and norms that promote a positive and productive work environment (teamwork, collaboration, encouragement of creativity and innovation, open and transparent communication, focus on personal growth and development and flexibility) while *Passive-defensive* with sub-cultures(approval, conventional, dependent and avoidance) is characterized by behaviours and norms that prioritize the avoidance of conflict and the preservation of the status quo. Employees in these cultures tend to conform to rules and procedures rather than challenging them. Key Features are avoiding mistakes and conflict, strictly following rules and procedures, tendency to seek approval and avoid risk, reliance on authority for decision-making and resistance to change and innovation. *Aggressive-defensive* (sub-cultures:oppositional,power,competitiveand,perfectionistic) is characterized by behaviours and norms that prioritize control, competition, and the protection of one’s interests. Employees in these cultures may be more focused on personal gain or competition with others than on collaboration and teamwork [55].

This study did not clearly indicate the prevailing OC in Egypt’s hospitals and its effect on WRS. However, very high levels of WRS were recorded and related to educational level, lack of leadership support, nurses’ capabilities, and external locus of control [55].

#### Positive and negative organizational cultures

Seven of our included studies [1, 5, 40, 57, 60, 61, 63] identified two main types of OC: positive and negative OC. Shirey et al., [40] highlights that features of positive OC include authentic leadership. A leader characterized by (purpose, values, heart, self-discipline) with behaviours of (empathy, respect, trust, and connectedness, balance) that are present in everyday work. Authentic leaders exert influence by engaging nurses rather than control and intimidation. Positive OC also has high support and empowerment of nurses, respect for nurses, effective communication throughout the organization, true collaboration and teamwork, nurses’ involvement in effective decision-making, adequate and appropriate staffing levels and regular meaningful recognition of nurses. In contrast, a negative OC lacks these qualities. Nurses experience limited support and empowerment, a lack of respect, poor communication, insufficient collaboration and unity, exclusion from decision-making processes, inadequate staffing and resources, and little or no recognition for their efforts. Such conditions can undermine job satisfaction, increase WRS, and negative impact on patient care [64, 65]. The other studies [1, 5, 57, 60, 61, 63] further explored and identified features of positive OC to include: effective clear communication and information flow, nurses involvement in leadership, governance and key decision making processes, high organizational and supervisors’ support, job clarity, high opportunities for education and professional development, advancement opportunities for career development, high inter-intra cadre collaboration and teamwork, individualized nurse’ need focus, continuous on job trainings, colleagues support, nurses’ respect, job and schedule predictability and flexibility, high levels of feeling valued, continuous professional development, professional recognition and respect, positive interpersonal relationships, manageable workloads, staff and resources adequacy, high internal locus of control and high levels of satisfaction (personal, professional and renumeration). Negative OC lacks the features of positive OC and both cultures are directly opposite of one another.

Studies from organizations that observed and maintained values of positive OC had low or moderately low levels of work-related stress while organizations that had features of negative OC had high levels of WRS. Positive OC also increased job satisfaction, nurses’ work engagements, and nurses’ retention while reducing burnout, emotional exhaustion, work-related stress and turnover intentions. Leading international organizations such as WHO [6],NIOSH [7, 8],ILO [9], ICN [10], EU-OSHA [11], ICOH [12] and ANA [14] strongly promote and advance these values of positive OC through comprehensive policy frameworks to improve nursing and healthcare.

#### Patient safety culture

Patient safety culture is defined by WHO and Agency for Healthcare Research and Quality [66] as “the absence of preventable harm to a patient and reduction of risk of unnecessary harm associated with health care to an acceptable minimum.” It included attitudes, perceptions, and values that employees have in relation to patient safety and focuses on the prevention of medical errors, surgical errors, healthcare-associated infections and sepsis, diagnosis errors, patient falls, venous thrombo-embolism, pressure ulcers, unsafe transfusion practices, patient misidentification and unsafe injection practices [49, 67, 68].

An organization’s patient safety culture is the extent to which an organization/hospital supports and promotes a patient safety culture. Its features are managers’ dedication to patient safety, manageable staff workloads, error reporting, teamwork, non-punitive policies, an open climate in the institute, and information exchange [49, 67, 68].

Habibzadeh et al., [62], in this review found that patient safety culture was negatively related to the Second Victim Experience (SVE) of stress (β = − 0.39, p < 0.01) and burnout (β = − 0.24, p < 0.01). The term “second victim” refers to healthcare workers who have been affected by unexpected unfavourable patient safety occurrences for example patient near-miss adverse effects. The patient is the first victim, and the healthcare worker is the second victim. When such errors occur, not only the patient suffers but also the nurse. The nurse also experiences emotional trauma, low self-confidence, and self-mistrust [68].

### Organizational Climate and Impact on Work-Related Stress

The general environment, work mood, and perception and view of work elements such as leadership, culture, and working conditions inside a healthcare institution or nursing organization are referred to as organizational climate in nursing. Two of our included studies [56, 58] studied organizational climate as an aspect of OC. Atabey et al., [55] found that when nurses perceive that their organization is dominated by unfavourable rules and high organizational focus rather than stakeholder’s and individual nurse focus, WRS increases. However [58], found that a positive appraisal of organizational climate (positive collaboration, respectful communication, non-conflictual communication, and more internal locus of control (belief that employees have control over problem-solving) strongly reduced WRS, increased self-esteem and doubled intention to stay within an organization and nursing profession.

### Organizational politics as an aspect of organizational culture and impact on work-related stress

Organizational Politics (OP) is a component of the organizational setting and nursing is not exceptional [59]. OP is a double-edged sword within the workplace. On one hand and the positive side, it is a dynamic force that influences decision-making, allocation of resources, and even career progression. It can unite people especially when it is used to promote fairness, transparency, and collaboration, the workplace becomes more harmonious.

On the other hand, it can be a significant source of work-related stress when wielded negatively. Conversely, positive organizational politics can alleviate stress and foster a healthier work environment. Negative OP thrives on pay and promotion policies that are tainted with favouritism, discrimination, disregard of merit, corruption, the existence of powerful few in the system, people you can’t challenge without repercussions, silencing critics, non-teamwork and non-collaboration of nurses, tense environment, and politics of destruction where people build themselves by tearing others down.

Movahedi et al., [59] found that on a Likert scale,5 indicating a higher satisfaction, perception of OP mean was 2.63. Correlation and Regression results of impact of positive OP were Job satisfaction (R^2^ = 0.219, β = 0.375, *P* = < 0.001), Job Stress (R^2^ = 0. 225, β = −0.384, *P* = < 0.001), Turnover (R^2^ = 0. 077, β = −0.322, *P* = < 0.001) and burnout (R^2^ = 0. 080, β = −0.506, *P* = < 0.001). These results indicate that positive perception of OP in Iran positively related to and increased job and pay, reduced WRS, burnout, and turnover intention.

## Discussion

To the best of our knowledge, this is the first systematic literature review attempting to relate the impact of OC on WRS among nurses. The aim of the study was to collect and collate scientific evidence in the literature, on the impact of OC on WRS among nurses and provide valuable insights into mitigation of WRS in nursing.

The results of this comprehensive review shed light on the intricate relationship between OC and WRS within the nursing and healthcare sectors. The analysis revealed three key thematic areas: types of OC, organizational climate, and organizational politics, each exerting a distinct impact on nurses’ work-related stress.

Diverse types of OC were highlighted. Bakertzis et al. (2020) identified four primary types of OC: Clan culture, Market culture, Hierarchy culture, and Adhocracy culture which were similarly identified by previous nursing studies [43, 44, 69, 70]. This study showed that the Greek healthcare sector was dominated by a hierarchical culture (prioritizes hierarchy, bureaucracy, efficiency, safe work operations, rule and order while focusing on organizational stability) and clan culture (working together like one large family, emphasizes teamwork, trust, respect, mentoring, participatory leadership, unity, and collaboration). The study found that clan and hierarchy culture tremendously decreased WRS and burnout. There was also high work engagement and job satisfaction among nurses. These results were similar to those of a study conducted in South Korea [71] which indicated that when nurses’ work relationships are healthy and positive, WRS and burnout are reduced. However, contrasted with [43] who had found that hierarchy culture was related to nurses’ turnover and stress. This implicated that with proper leadership and work “clannism”, WRS can extensively be reduced.

Moustafa Manal Saleh et al. (2014) classified OC into three main categories: Constructive, Passive-defensive, and Aggressive-defensive OCs. This classification is also similar to those of other studies [72, 73]. Constructive cultures emphasize positive and productive behaviours that enhance the work environment, such as teamwork, collaboration, creativity, and innovation. On the other hand, Passive-defensive and Aggressive-defensive cultures prioritize avoiding conflict and personal gain, respectively, potentially at the expense of collaboration and teamwork. Constructive culture is associated with low WRS and high job satisfaction, while negative cultures, particularly aggressive-defensive ones, contribute to high levels of WRS, burnout, and turnover intentions [72]. In this study, nurses approved that nursing organizations should have constructive OC; however, it was not the case and WRS was extremely high.

The climax and epitome of this review was the classification of OC into positive and negative cultures. This was the commonest classification among the included studies [1, 5, 40, 57, 60, 61, 63]. A positive culture is characterized by several values: effective clear communication and information flow, nurses’ involvement in leadership, governance and key decision-making processes, high organizational and supervisors’ support, job clarity, high opportunities for education and professional development, advancement opportunities for career development, high inter-intra cadre collaboration and teamwork, individualized nurse’ need focus, continuous on the job training, colleagues support, nurses’ respect, job and schedule predictability and flexibility, high levels of feeling valued, continuous professional development, professional recognition and respect, positive interpersonal relationships, manageable workloads, staff and resources adequacy, high internal locus of control and high levels of satisfaction (personal, professional and remuneration). These factors were identified from seven of our included studies and are referred to as perfect OC values to mitigate WRS. Negative OC, in contrast, lacked these supportive elements and contributed to increased stress and burnout among nurses. The difference is stark, with organizations that maintain the values of positive cultures experiencing lower work-related stress levels [5, 40, 58, 60, 61, 63], higher job satisfaction, and enhanced nurse retention. It is evident that the presence or absence of these cultural attributes significantly impacts the well-being of nursing professionals. These results are also firmly affirmed by other studies [3, 4, 16, 21, 74–76] who found the same results.

Organizational climate encompasses the overall perception and view of the work environment, leadership, culture, and working conditions, rules, organizational and leadership support among other aspects of the organization. Employees’ perceptions and appraisals can be positive or negative and this notable impact on their WRS. Studies conducted by Atabay et al., [56] and Siket Ujváriné et al., [58] highlight the significance of nurses’ perceptions in shaping their experiences. Unfavourable rules, a high organizational focus rather than individualized nurse focus, and a lack of focus on stakeholder (nurses, doctors and health workers) well-being in the organization can increase organizational cynicism which in turn contributes to increased WRS. On the other hand, a positive perception of the organizational climate, characterized by collaborative, respectful communication, non-conflictual interaction, and an internal locus of control, can significantly reduce WRS, boost self-esteem, and increase nurses’ intention to remain within the organization and the nursing profession. Organizational climate is a critical aspect of OC. Apart from influencing WRS, when positive, can enhance organizational commitment, job satisfaction, and work engagement, improve work performance, reduce nurses’ turnover intention, reduce absenteeism as well as increase collaboration among workers [48, 77–82].

Organizational politics is defined as “an individual’s deliberate activities focused towards achieving one’s own self-interests while disregarding the interests and welfare of others or their organization” [4, 83]. This review acknowledges the existence of negative and positive OP. Negative OP is marked as wielding power negatively, characterized by favouritism, discrimination, corruption, and the presence of powerful individuals who are beyond challenge within the nursing work environment. The mighty and powerful deep state of a selected few. This undermines teamwork and professional autonomy, creating a tense environment of distrust and stressful work environment [84]. It also creates a dependency culture on OP and polarizes hospital and nursing work environments.

In contrast, positive OP can foster a positive work environment characterized by strong collaboration and teamwork among nurses and, a transparent workplace where merit is exercised in access to promotions and employment practices. Discrimination against race, gender, tribe, sexual orientation, colour, transfers, promotions and all its forms are also lowered. In this review, Movahedi et al. [59] reveal that a positive perception of OP in Iran is associated with increased job and pay satisfaction, reduced job stress, burnout, and turnover intention. These results are also echoed by [4, 84, 85].

### Limitations

The majority of the studies considered in this review were published in English as free access papers. Articles from other languages were not included and, in the process, useful information might have been excluded. Conference papers, other systematic reviews and meta-analyses of the literature, letters and opinions, editorials, essays, case studies, and literature, comments, research including mixed nurses and other cadres were also omitted. As a result, crucial information e.g. for comparing the impact may have been left out.

## Conclusions

This comprehensive review represents one of pioneering efforts to investigate the relationship between organizational culture and WRS among nurses. The study aimed to compile and analyse the existing scientific evidence on this subject to offer valuable insights for addressing work-related stress in nursing.

Our findings illuminate the intricate connection between OC and WRS within the nursing and healthcare sectors. The analysis identified three significant thematic areas: types of OC, organizational climate, and OP, each exerting distinct influence on nurses’ WRS. The review confirms and re-affirms and unequivocally establishes that a positive organizational culture, positive organizational climate, and positive organizational politics are not merely advantageous but indispensable within the nursing profession. They stand as resolute solutions, beacons of transformation for nursing work environments, and unwavering shields against the relentless tide of WRS.

Therefore, nurse managers, leaders and all stakeholders should be aware of OC and thrive always to build positive culture, climate and politics within nursing work environments. Policy frameworks on positive OC, OP and organizational climate developed and advocated by internationally leading authorities such as WHO, NIOSH, ILO, ANA, EU-OSHA, ICOH and FOHNEU, aimed at cultivating positive work environment for nurses and other medical professionals should be vigorously adopted and implemented across all healthcare settings. Embracing these best practices not only enhances staff well-being but also directly correlates with improved patient outcomes, retention rates, and work resilience, which are critical in addressing global nursing and healthcare problems.

## Supplementary Information

Supplementary Material 1.

Supplementary Material 2.

## Data Availability

Data is provided within the manuscript and supplimentary files. Further datasets supporting the conclusions of this review are not publicly available but are available from the corresponding author on reasonable request.

## Abbreviations

ANA: American Nurses Association
CINAHL: Cumulative Index to Nursing and Allied Health Literature
OC: Organizational Culture
EU-OSHA: European Agency for Safety and Health at Work
FOHNEU: Federation of Occupational Health Nurses within the European Union
ILO: International Labour Organization
ICN: International Council of Nurses
ICOH: International Commission on Occupational Health
MMAT: The Mixed Methods Appraisal Tool
NIOSH: National Institute for Occupational Safety and Health
OCI^®^: Organizational Culture Assessment Instrument
OP: Organizational Politics
PEO: Population Exposure Outcome
PRISMA: Preferred Reporting Items for Systematic Reviews and Meta-Analysis
US$: United States Dolars
WHO: World Health Organization
WRS: Work-Related Stress
