# Supplementary material for: Transforming nursing work environments: the impact of organizational culture on work-related stress among nurses: a systematic review
Source: BMC Health Serv Res. 2024 Dec 2;24:1526. doi: 10.1186/s12913-024-12003-x (PMC11613752; doi:10.1186/s12913-024-12003-x)
Supplement: Supplementary file 2 — Supplementary Material 2. [file 12913_2024_12003_MOESM2_ESM.docx]

| **Study** | **TABLE S1 SUPPLIMENTARY:**  **Quality appraisal using the Mixed Methods Appraisal Tool MMAT (n = 13)** | | | | | | | | | | | | | | | | | | | | | | | | | Quality |
| --- | --- | --- | --- | --- | --- | --- | --- | --- | --- | --- | --- | --- | --- | --- | --- | --- | --- | --- | --- | --- | --- | --- | --- | --- | --- | --- |
|  | 1.1 | 1.2 | 1.3 | 1.4 | 1.5 | 2.1 | 2.2 | 2.3 | 2.4 | 2.5 | 3.1 | 3.2 | 3.3 | 3.4 | 3.5 | 4.1 | 4.2 | 4.3 | 4.4 | 4.5 | 5.1 | 5.2 | 5.3 | 5.4 | 5.5 |  |
| B. Papadionysiou et al.,2022 |  |  |  |  |  |  |  |  |  |  |  |  |  |  |  | 0 | 0 | 1 | 1 | 1 |  |  |  |  |  | ******* |
| Manal et al.,2014 |  |  |  |  |  |  |  |  |  |  |  |  |  |  |  | 1 | 1 | 1 | 0 | 1 |  |  |  |  |  | ******** |
| Atabay et al.,2014 |  |  |  |  |  |  |  |  |  |  |  |  |  |  |  | 1 | 1 | 1 | 1 | 1 |  |  |  |  |  | ********* |
| Van et al.,2014 |  |  |  |  |  |  |  |  |  |  |  |  |  |  |  | 1 | 1 | 1 | 1 | 1 |  |  |  |  |  | ********* |
| Siket et al,.2020 |  |  |  |  |  |  |  |  |  |  |  |  |  |  |  | 1 | 1 | 1 | 1 | 1 |  |  |  |  |  | ********* |
| Shirey et al.,2009 | 1 | 1 | 1 | 1 | 1 |  |  |  |  |  |  |  |  |  |  |  |  |  |  |  |  |  |  |  |  | ********* |
| Movahedi et al,.2020 |  |  |  |  |  |  |  |  |  |  |  |  |  |  |  | 1 | 1 | 1 | 1 | 1 |  |  |  |  |  | ********* |
| Lee et al., 2020 |  |  |  |  |  |  |  |  |  |  |  |  |  |  |  | 1 | 1 | 1 | 1 | 1 |  |  |  |  |  | ********* |
| Hayes et al.,2015 |  |  |  |  |  |  |  |  |  |  |  |  |  |  |  | 1 | 1 | 1 | 1 | 1 |  |  |  |  |  | ********* |
| Hahtela et al.,2015 |  |  |  |  |  |  |  |  |  |  |  |  |  |  |  | 1 | 1 | 1 | 1 | 1 |  |  |  |  |  | ********* |
| Habibzadeh et l.,2020 |  |  |  |  |  |  |  |  |  |  |  |  |  |  |  | 1 | 1 | 1 | 1 | 1 |  |  |  |  |  | ********* |
| Eskola 2016 et al., |  |  |  |  |  |  |  |  |  |  |  |  |  |  |  | 1 | 0 | 1 | 0 | 1 |  |  |  |  |  | ******* |
| Aghaei et al.,2020 |  |  |  |  |  |  |  |  |  |  |  |  |  |  |  | 0 | 1 | 1 | 1 | 1 |  |  |  |  |  | ******** |

5***** or 100% quality criteria met, 4**** or 80% quality criteria met, 3*** or 60% quality criteria met, 2** or 40% quality criteria met and 1* or 20% quality criteria met.
